# Effectiveness of cervical cancer screening at age 65 — A register-based cohort study

**DOI:** 10.1371/journal.pone.0214486

**Published:** 2019-03-26

**Authors:** Maiju Pankakoski, Ahti Anttila, Tytti Sarkeala, Sirpa Heinävaara

**Affiliations:** 1 Finnish Cancer Registry, Helsinki, Finland; 2 Faculty of Medicine, University of Helsinki, Helsinki, Finland; Rudjer Boskovic Institute, CROATIA

## Abstract

Most cervical cancer deaths in Finland occur after the termination of the national screening program, targeted at women aged 30 to 64 years. The purpose of this study was to examine the effectiveness of screening at age 65 in reducing cervical cancer mortality. A register-based cohort study was performed with a follow-up period between 1991 and 2014. Mortality risk ratios for incident cervical cancer cases diagnosed at age 65 or older were compared between women invited and not invited for screening. The background risk difference between the studied areas was accounted for by using a reference cohort. The relative risk of death for women invited for cervical cancer screening at the age of 65 was 0.52 (95% CI: 0.29–0.94). The relative risks for women not attending and attending to screening with respect to the uninvited were 1.28 (CI: 0.65–2.50) and 0.28 (CI: 0.13–0.59), respectively. Inviting 65-year-old women for screening has been effective in reducing cervical cancer mortality.

## Introduction

There is no clear evidence on the appropriate age at which to stop screening for cervical cancer [1]. The organized cervical cancer screening program in Finland, mainly targeted to women aged 30–64 years, has been shown to be the most effective among women above the age of 40 [2]. However, an increasing number of studies have suggested that screening also women aged 65 and older reduces cancer incidence and mortality [3–5].

Cervical cancer is caused by the human papillomavirus (HPV) infection, and the probability of acquiring a new infection is the highest at a young age [6]. The decreasing HPV-prevalence by age is often considered to justify the discontinuation of screening older women. Cervical cancer incidence is also relatively small in elderly women with a history of negative screening results. Castañón et al. demonstrated that stopping the screening of women with, for example, three or more consecutive negative screening results before age 65 could be safe [7]. However, it has also been demonstrated that the risk for cervical cancer after consecutive negative screening results is similar for women aged 50 and for women of younger ages [8]. Therefore, a history of negative results at an older age might not be a sufficient reason to stop screening.

The growing life expectancy is likely to effect the future of cervical cancer burden among the elderly. Although cervical cancers are relatively rare among older women who have been offered screening earlier in life, they tend to be more severe with a poorer prognosis compared to cancers among the young [9]. Thus, early detection of cancers in the older population would be beneficial, reducing disease burden and mortality.

The aim of this study was to determine whether inviting women aged 65 to screening is associated with reduced mortality from cervical cancer.

## Materials and methods

The national target age for cervical cancer screening in Finland is 30–60, which corresponds to the minimum target age range recommended by the European screening guidelines [1]. Screening invitations are sent every five years to women in the target age range. Follow-up screening 1–2 years after the routine screen is recommended for women with mild abnormalities. These screenings continue until the next screening round (or age 64) if necessary. The screening test is mostly conventional cytology. However, primary HPV testing with a cytology triage was used in some areas as a part of a randomized trial in 2003–2012 [10]. Since 2012, primary HPV testing has been incorporated into the cervical cancer screening program in an increasing number of areas [11].

Individual municipalities are responsible for the screening organization and the majority of them stop screening at the minimum required age of 60. However, some municipalities continue inviting women until age 65. The capital city of Helsinki has invited all 65-year-old women regularly since 1991 [12]. This enabled us to study the effectiveness of screening at age 65 by comparing women invited to screening in Helsinki to those who were not invited elsewhere. The invited population was limited to Helsinki only since the background risk of cervical cancer was considerably higher in the capital city, also among the older age groups [13]. In addition, only 20% of the invited women were living outside Helsinki, and they were allocated sparsely and invited irregularly during the follow-up. Thus, the invited women were all residents of Helsinki, whereas the uninvited were all from other parts of the country.

We performed a population-based cohort study with a follow-up period in 1991–2014. A cohort of approximately one million women born in 1926–1956 and aged 55–65 at the beginning of the follow-up was derived from the population registry. Cervical cancer diagnoses and deaths from incident cases in women aged 55 and older until the year 2014 were linked from the cancer registry. The cohort’s screening records from 1991–2011 were then linked from the Mass Screening Registry.

**Fig 1:**
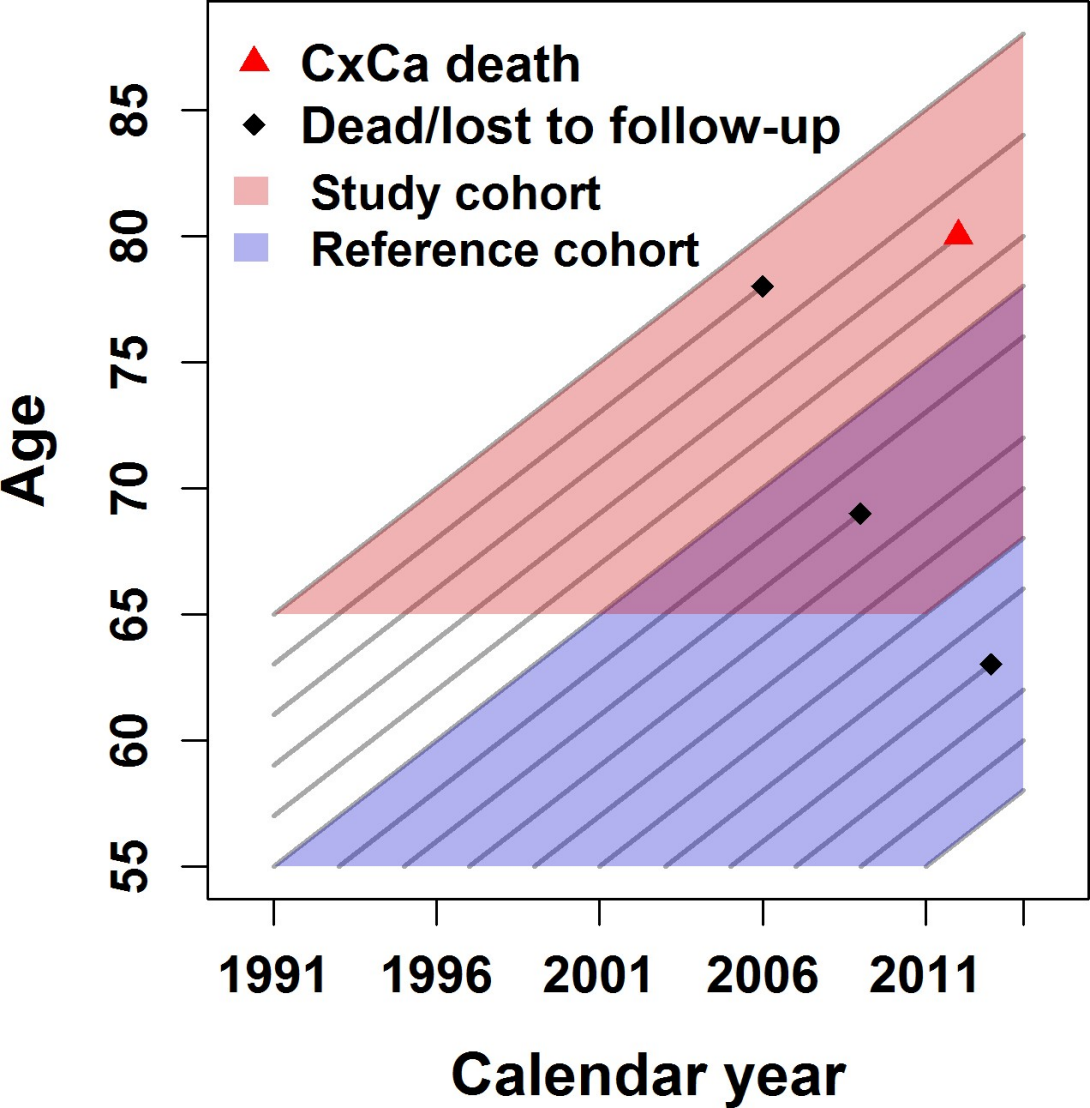
Illustration of the events and person-years in the study and reference cohorts. In the reference cohort, cervical cancer (CxCa) deaths after age 55 were considered for incident cancer cases diagnosed at ages 55–64. In the study cohort, CxCa deaths were considered for incident cancer cases diagnosed at age 65 or older.

**Fig 2:**
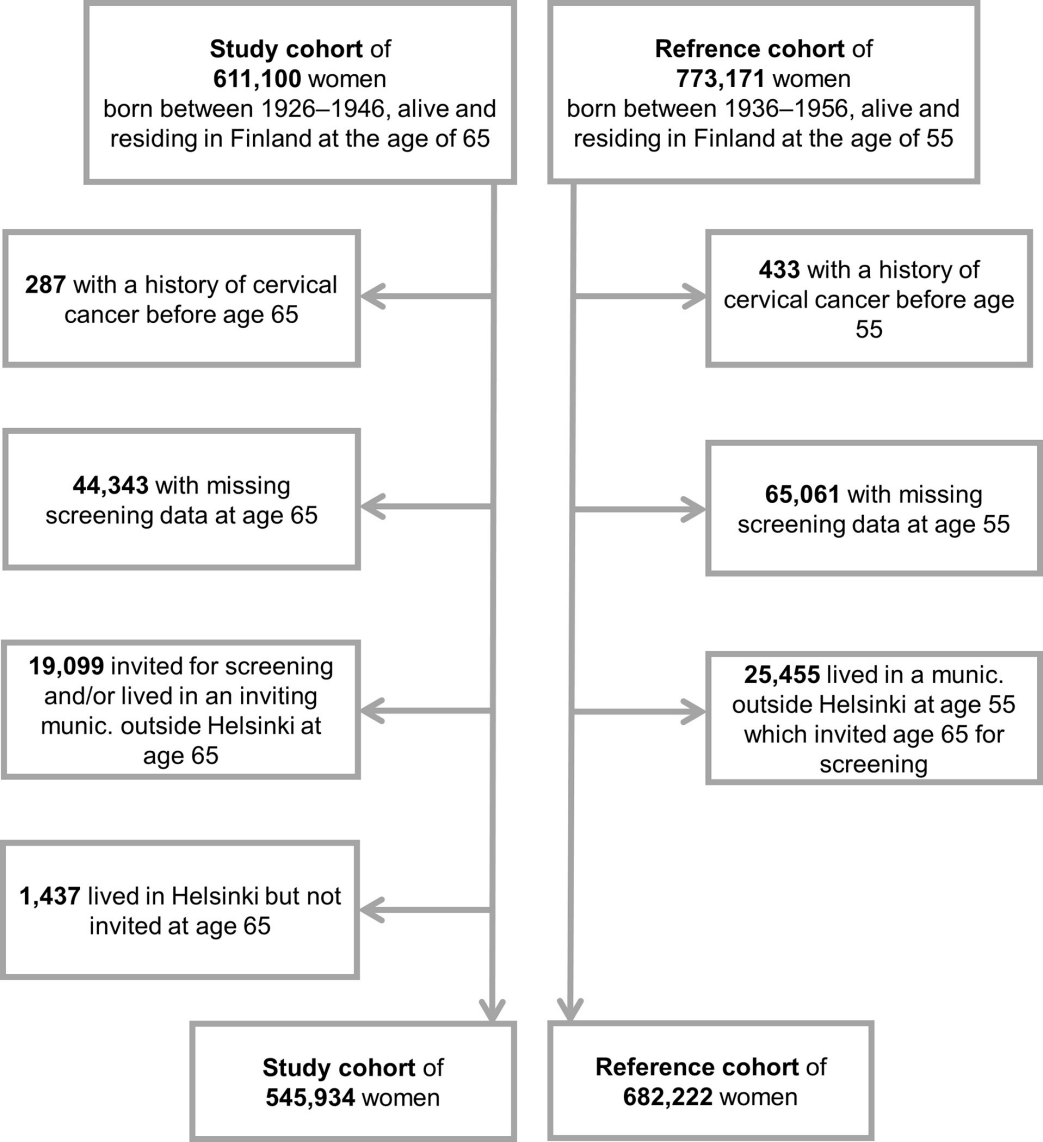
Exclusions from the total cohort, including the study and reference cohorts. An individual woman might have contributed to both the study and reference cohorts. Follow-up between 1991 and 2014.

Data on deaths due to cases diagnosed at age 55–64 were used to assess the background risk difference between Helsinki and the comparison regions (*a reference cohort* of women born in 1936–1956, N = 773,171). We considered ten years as an appropriate minimum time for the cancer incidence follow-up. Deaths from incident cases at age 65 years or older were used to assess the association between an invitation at age 65 and cervical cancer mortality (*a study cohort* of women born in 1926–1946, N = 611,100). An individual woman might have contributed to both the study and reference cohorts. The research data are illustrated in Fig 1. After exclusions e.g. due to previous cervical cancers and missing screening data, we ended up with a total cohort of 954,128 women. Data exclusions are shown in more detail in Fig 2.

We estimated the incidence-based mortality risk ratio of cervical cancer for women invited to routine screening at the age of 65 compared to those not invited. The risk ratio was calculated using Poisson regression. Person-years (median = 11.1) were recorded until death, emigration or end of follow-up period. An interaction term between the cohort indicator (study vs. reference cohort) and the residential area was included in the model. The term provided us with a background risk adjusted effectiveness estimate of invitation at age 65 in the study cohort. Risk ratios were also estimated for attending and non-attending women with respect to the uninvited.

The use of confidential registries was approved by the National Institute for Health and Welfare (permit no. 735/5.05.00/2015). Statistical analyses were performed using the R program (version 3.5.1) [14].

## Results

Of the women in the study cohort, 11% lived in Helsinki and received an invitation for routine screening at the age of 65 and around 75% of them attended. In the reference cohort, around 12% lived in the city of Helsinki at the beginning of the follow-up (Table 1). Among these women, the risk of death from cervical cancer was elevated (RR = 1.91, 95% CI: 1.25–2.92), compared to those living in municipalities not inviting 65-year-olds. This estimate was considered as a measure of the geographical background risk difference between Helsinki and the areas not inviting age 65.

**Table 1:** Characteristics and unadjusted cervical cancer mortality rates (per 100,000 person-years) by invitational status / residential area in the study and reference cohorts.

**Study cohort: women born in 1926–1946**	**N (%)**	**Birth year (mean)**	**CxCa deaths^a^**	**100 000 Person-years**	**Rate per 100 000**
**All at age 65**	545,934 (100)	1937.2	237	62.3	3.8
**Not invited**	486,869 (89)	1937.1	212	55.7	3.8
**Invited (Helsinki)**	59,065 (11)	1937.3	25	6.6	3.8
**Did not attend**	15,054 (3)	1936.8	15	1.6	9.3
**Attended**^**c**^	44,011 (8)	1937.4	10	5.0	2.0
**Reference cohort cohort: women born in 1936–1956**	**N (%)**	**Birth year (mean)**	**CxCa deaths**^**b**^	**100 000 Person-years**	**Rate per 100 000**
**All at age 55**	682,222 (100)	1947.5	130	81.7	1.6
**Lived in a munic. which did not invite age 65**	600,937 (88)	1947.5	103	71.9	1.4
**Lived in a munic. which invited age 65**	81,285 (12)	1947.1	27	9.9	2.7

CxCa, cervical cancer; munic., municipality.

^a^ Deaths for cervical cancers diagnosed at age 65 or older.

^b^ Deaths for cervical cancers diagnosed at ages 55–64.

^c^ Attendance rate 75%.

The background risk adjusted relative risk of death from cervical cancer for women invited at the age of 65 was 0.52 (95% CI: 0.29–0.94), compared to the uninvited. The relative risks for women not attending and attending to screening with respect to the uninvited were 1.28 (95% CI: 0.65–2.50) and 0.28 (95% CI: 0.13–0.59), respectively (Table 2).

**Table 2:** Poisson model estimates for the effect of screening invitation in Helsinki and attendance at age 65 on cervical cancer mortality.

	crude RR^a^	adjusted RR^b^
**Not invited**	1	1
**Invited**	1.00 (0.66–1.51)	0.52 (0.29–0.94)
**Did not attend**	2.44 (1.45–4.12)	1.28 (0.65–2.50)
**Attended**	0.51 (0.27–0.96)	0.28 (0.13–0.59)

^a^ Only study cohort analysed.

^b^ Study and reference cohorts combined and risk ratios adjusted for the background risk difference between the areas.

Descriptive results on the screening history before age 65 among women in the study cohort are presented in Table 3. The crude mortality rate for cervical cancer deaths diagnosed after age 65 was lower for women attending to screening at ages 55–64 compared to non-attending women or women without available information on previous screening history. In Helsinki, where also 65-year-olds were invited, a larger proportion of women had been screened at ages 55–64 compared to the rest of the country. The mortality rate was higher also among previously screened women (2.4 vs. 1.6), although number of cases was relatively small.

**Table 3:** Characteristics and unadjusted cervical cancer mortality rates (per 100,000 person-years) by invitational status at age 65 and screening history at ages 55–64 in the study cohort.

Study cohort: women born in 1926–1946	N (%)	Birth year (mean)	CxCa deaths^a^	100 000 Person-years	Rate per 100 000
**Not invited at age 65**	486,869 (100)	1937.1	212	55.7	3.8
** Not screened at 55–64**	122,926 (25)	1937.5	72	14.1	5.1
** Screened at 55–64**	252,750 (52)	1940.8	36	22.0	1.6
** Screening history not known**	111,193 (23)	1928.5	104	19.7	5.3
**Invited at age 65 (Helsinki)**	59,065 (100)	1937.3	25	6.6	3.8
** Not screened at 55–64**	9,525 (16)	1938.5	5	1.0	5.2
** Screened at 55–64**	36,658 (62)	1940.0	8	3.4	2.4
** Screening history not known**	12,882 (22)	1928.5	12	2.2	5.4

CxCa, cervical cancer

^a^ Deaths for cancers diagnosed at age 65 or older.

Out of all 237 cervical cancer deaths in the study cohort, 76 (32%) were due to adenocarcinomas. Other cancer types are shown in Table 4.

**Table 4:** Cancer types in the study cohort for cancers diagnosed at age 65 or older (N = 237).

Study cohort: women born in 1926–1946	N (%)
**Squamous cell carcinoma**	130 (55)
**Adenocarcinoma**	76 (32)
**Carcinoma other or unspecified**	24 (10)
**Other**	7 (3)

## Discussion

We observed an almost 50% reduction in cervical cancer mortality among women invited to screening in the city of Helsinki at age 65 compared to the uninvited women living elsewhere. For attendees, the corresponding mortality reduction was over 70%. However, a part of the result among the attendees is explained by self-selection, that is, the selection of women in the screened group who are already at lower risk of cervical cancer. Women who did not attend had an elevated risk of cervical cancer compared to the uninvited. Therefore, screening at age 65 might be even more effective if we were able to improve the attendance rate.

The challenge was to account for the different background risks between the capital city of Helsinki and the municipalities not inviting 65-year-olds. Helsinki had a more extensive screening target age group (25–65) during the entire follow-up but also the highest background risk of cervical cancer. In many high income countries, cervical cancer risk has found to be elevated in urban areas [15,16]. Therefore, the wider target age range of cervical cancer screening in Helsinki has undoubtedly been beneficial in terms of mortality reduction, although the unadjusted differences between the invited and uninvited women were obscured by the higher background risk compared to the rest of the country. Our descriptive results indicated that the risk of death from cervical cancer after age 65 was higher in Helsinki also among women who had been screened at ages 55–64.

A relatively high percentage of the deaths in the study cohort were due to adenocarcinomas. In the future, HPV testing might be especially useful for detecting adenocarcinomas, which are less likely to be prevented by conventional cytology [17]. Only a minority of the Finnish municipalities are currently using HPV testing as the primary screening method. During the study period, also Helsinki was mostly using cytology screening. However, HPV based screening is now becoming more widespread, and is being introduced in Helsinki in 2019. It will be interesting to see whether this will have an effect on cervical cancer mortality, specifically on deaths due to adenocarcinoma.

This was the first time that the effectiveness of screening at age 65 was studied in Finland using longitudinal cohort data with an extensive follow-up time of 24 years. Since we compared the invited women to the uninvited, we were able to see the impact of screening at the population level without having to tackle the problem of self-selection bias. Statistical power was, however, somewhat limited due to the rareness of cervical cancer deaths and the relatively small proportion of women invited in the oldest age group. However, our results are in line with previous studies reporting reduced mortality rates for women screened until the age of 69. An earlier case-control study from Finland found a long-lasting protective effect of screening at ages 55–69 and a strong indication of additional benefit from extending the upper age to 65 [4]. Similar results were also reported for example in studies from the US and Canada [3,5]. A nationwide extension of the upper age of screening would also be cost-effective. An earlier modelling study showed that screening until age 70 was cost-effective with 8450 euros per QALY [18].

Cervical cancer incidence has decreased substantially in Finland after the beginning of screening in the early 1960s [19]. Since then, the decreasing trend has been consistent among older ages due to the ever-increasing number of older women having been exposed to screening. The higher incidence at an older age could at least partly be explained by the screening histories of the oldest birth cohorts [20,21]. In the study cohort the oldest women had their first screen around the age of 40 in the late 1960s, and thus had a smaller number of lifetime screens than the younger women. It is safe to assume that all women in our cohort have been subjected to a similar screening policy at least since their 40s or 50s. Even so, it remains to be seen whether the incidence and mortality of women over the age of 65 will be different in the future when the lifetime screening histories will be more similar across birth cohorts.

The hysterectomy prevalence was presumably quite high in our study cohort. Unfortunately, we were not able to have individual-level data on hysterectomies in this study. Luoto et al. (2004) estimated that approximately one-fifth of women aged 45–64 have undergone a hysterectomy in Finland. A peak in the rate was observed in the birth cohort born in 1943–47, after which it started to decline [22]. However, it is unlikely that the hysterectomy prevalence has differed significantly between Helsinki and the rest of the country, thanks to the unified current care guidelines since 1994 [23]. Even if differences existed, we assume that our model takes also this into account.

In this study, only tests taken within the organized screening program were available for analysis. Salo et al. (2014) estimated that the overall five-year coverage of Pap tests in Finland is as high as 87%, when also tests outside the organized program are taken into account. These opportunistic tests are taken especially from young women or women who are within the target age range of the program. The overall test coverage decreases substantially at older ages, after the termination of the program, when also the opportunistic testing is reduced [24]. However, if in Helsinki women of older ages would have had opportunistic tests more frequently than in other parts of the country, it could have also explained some part of the mortality difference. Incorporating also opportunistic data would be an asset in the future.

In 2017 the average life-expectancy of a Finnish woman at birth was approximately 84 years, and 24 percent of the female population was aged 65 and older [25,26]. The relative size of the older population has grown over the more recent years across the world, as well as the prevalence of cervical cancer risk factors, such as increased sexual activity [27,28]. At the moment most of the deaths from cervical cancer in Finland occur after the last screening invitation [4], when also Pap testing outside the organized program decreases substantially. Therefore, it is likely that the cervical cancer burden among the older population will not lessen in the near future.

To conclude, our analysis showed a significant cervical cancer mortality reduction among women invited for screening in Helsinki at the age of 65. The extension of the organized screening program to an older age in the capital city has thus been beneficial. These results could be generalizable to other countries and regions with long-standing and good-quality screening programs. Several studies have already confirmed the increased risk of cervical cancer in older women with earlier abnormal or unavailable screening results [29–31]. Therefore, the continuation of screening for cervical cancer until a sufficiently old age should be ensured.

## References

[1] Arbyn M, Anttila A, Jordan J, Ronco G, Schenck U, Segnan N, et al European Guidelines for Quality Assurance in Cervical Cancer Screening. Second Edition—Summary Document. Ann Oncol. 2010;21:448–58. 10.1093/annonc/mdp471 20176693 PMC2826099

[2] Lönnberg S, Anttila A, Luostarinen T, Nieminen P. Age-Specific Effectiveness of the Finnish Cervical Cancer Screening Programme. Cancer Epidemiol Biomarkers Prev. 2012;21:1354–61. 10.1158/1055-9965.EPI-12-0162 22665576

[3] Rustagi AS, Kamineni A, Weinmann S, Reed SD, Newcomb P, Weiss NS. Cervical Screening and Cervical Cancer Death Among Older Women: A Population-Based, Case-Control Study. Am J Epidemiol. 2014;179:1107–14. 10.1093/aje/kwu035 24685531 PMC3992820

[4] Lönnberg S, Nieminen P, Luostarinen T, Anttila A. Mortality audit of the Finnish cervical cancer screening program. Int J Cancer. 2013;132:2134–40. 10.1002/ijc.27844 22987437

[5] Vicus D, Sutradhar R, Lu Y, Elit L, Kupets R, Paszat L. The association between cervical cancer screening and mortality from cervical cancer: A population based case–control study. Gynecol Oncol. 2014;133:167–71. 10.1016/j.ygyno.2014.02.037 24589414

[6] Smith JS, Melendy A, Rana RK, Pimenta JM. Age-Specific Prevalence of Infection with Human Papillomavirus in Females: A Global Review. Expand Our Knowl HPV Common Cause Cancers US Worldw. 2008;43:S5.e1–S5.e62. 10.1016/j.jadohealth.2008.07.009 18809145

[7] Castañón A, Landy R, Cuzick J, Sasieni P. Cervical Screening at Age 50–64 Years and the Risk of Cervical Cancer at Age 65 Years and Older: Population-Based Case Control Study. Franco EL, editor. PLoS Med. 2014;11:e1001585 10.1371/journal.pmed.1001585 24453946 PMC3891624

[8] Rebolj M, van Ballegooijen M, Lynge E, Looman C, Essink-Bot M-L, Boer R, et al Incidence of cervical cancer after several negative smear results by age 50: prospective observational study. The BMJ. 2009;338:b1354 10.1136/bmj.b1354 19395420 PMC2673344

[9] Darlin L, Borgfeldt C, Widén E, Kannisto P. Elderly Women Above Screening Age Diagnosed with Cervical Cancer Have a Worse Prognosis. Anticancer Res. 2014;34:5147–51. 25202106

[10] Anttila A, Hakama M, Kotaniemi-Talonen L, Nieminen P. Alternative technologies in cervical cancer screening: a randomised evaluation trial. BMC Public Health. 2006;6:1–8. 10.1186/1471-2458-6-1 17042938 PMC1621071

[11] Finnish Cancer Registry. Screening statistics [Internet]. 2017 [cited 2018 May 21]. Available from: https://cancerregistry.fi/statistics/screening-statistics.

[12] Tarkkanen J, Geagea A, Nieminen P, Anttila A. Quality improvement project in cervical cancer screening: practical measures for monitoring laboratory performance. Acta Obstet Gynecol Scand. 2003;82:82–8. 12580846 10.1034/j.1600-0412.2003.820115.x

[13] Finnish Cancer Registry. Cancer statistics [Internet]. 2017 [cited 2018 May 21]. Available from: https://cancerregistry.fi/statistics/cancer-statistics.

[14] R Core Team. R: A Language and Environment for Statistical Computing [Internet]. Vienna, Austria: R Foundation for Statistical Computing; 2018 Available from: https://www.R-project.org/

[15] Sharp L, Donnelly D, Hegarty A, Carsin A-E, Deady S, McCluskey N, et al Risk of Several Cancers is Higher in Urban Areas after Adjusting for Socioeconomic Status. Results from a Two-Country Population-Based Study of 18 Common Cancers. J Urban Health Bull N Y Acad Med. 2014;91:510–25. 10.1007/s11524-013-9846-3 PMC4074316 24474611

[16] Siesling S, van der Aa MA, Coebergh JW, Pukkala E, null null. Time‐space trends in cancer incidence in The Netherlands in 1989–2003. Int J Cancer. 2008;122:2106–14. 10.1002/ijc.23358 18183593

[17] Castanon A, Landy R, Sasieni PD. Is cervical screening preventing adenocarcinoma and adenosquamous carcinoma of the cervix? Int J Cancer. 2016;139:1040–5. 10.1002/ijc.30152 27096255 PMC4915496

[18] Nieminen, P, Anttila, A, Apter, D, Grenman, S. Terveyden ja hyvinvoinnin laitoksen asettaman papilloomavirustautien torjuntatyöryhmän selvitys [Report by a working group set by the National Institute for Health and Welfare] (In Finnish). 2011. Report No.: 28.

[19] Hristova L, Hakama M. Effect of screening for cancer in the Nordic countries on deaths, cost and quality of life up to the year 2017. Acta Oncol Stockh Swed. 1997;36 Suppl 9:1–60. 9143316

[20] Lynge E, Lönnberg S, Törnberg S. Cervical cancer incidence in elderly women-biology or screening history? Eur J Cancer. 2017;74:82–8. 10.1016/j.ejca.2016.12.021 28335890

[21] Seppä K, Pitkäniemi J, Malila N, Hakama M. Age‐related incidence of cervical cancer supports two aetiological components: a population‐based register study. BJOG Int J Obstet Gynaecol. 2016;123:772–8. 10.1111/1471-0528.13754 26599730

[22] Luoto R, Raitanen J, Pukkala E, Anttila A. Effect of hysterectomy on incidence trends of endometrial and cervical cancer in Finland 1953–2010. Br J Cancer. 2004;90:1756–9. 10.1038/sj.bjc.6601763 15208619 PMC2409756

[23] Vainikainen T. Kuka tarvitsee Käypä hoito-suosituksia. Duodecim (In Finnish). 2003;119:1183–6.

[24] Salo H, Nieminen P, Kilpi T, Auranen K, Leino T, Vänskä S, et al Divergent coverage, frequency and costs of organised and opportunistic Pap testing in Finland. Int J Cancer. 2014;135:204–13. 10.1002/ijc.28646 24347441

[25] Statistics Finland. Official Statistics of Finland (OSF): Deaths. ISSN = 1798–2545. Helsinki [Internet]. 2018 [cited 2018 May 28]. Available from: http://www.stat.fi/til/kuol/index_en.html.

[26] Statistics Finland. Official Statistics of Finland (OSF): Population structure. ISSN = 1797–5395. Helsinki [Internet]. 2018 [cited 2018 May 28]. Available from: http://www.stat.fi/til/vaerak/tau.html.

[27] Beckman N, Waern M, Gustafson D, Skoog I. Secular trends in self reported sexual activity and satisfaction in Swedish 70 year olds: cross sectional survey of four populations, 1971–2001. BMJ [Internet]. 2008;337 Available from: http://www.bmj.com/content/337/bmj.a279.abstract 10.1136/bmj.a279 PMC2483873 18614505

[28] Lindau ST, Gavrilova N. Sex, health, and years of sexually active life gained due to good health: evidence from two US population based cross sectional surveys of ageing. BMJ [Internet]. 2010;340 Available from: http://www.bmj.com/content/340/bmj.c810.abstract 10.1136/bmj.c810 PMC2835854 20215365

[29] Blanks RG, Moss SM, Addou S, Coleman DA, Swerdlow AJ. Risk of cervical abnormality after age 50 in women with previously negative smears. Br J Cancer. 2009;100:1832–6. 10.1038/sj.bjc.6605069 19417745 PMC2695690

[30] Wang J, Andrae B, Sundström K, Ploner A, Ström P, Elfström KM, et al Effectiveness of cervical screening after age 60 years according to screening history: Nationwide cohort study in Sweden. PLOS Med. 2017;14:e1002414 10.1371/journal.pmed.1002414 29065127 PMC5655486

[31] Kotaniemi-Talonen L, Malila N, Anttila A, Nieminen P, Hakama M. Intensified screening among high risk women within the organised screening programme for cervical cancer in Finland. Acta Oncol. 2011;50:106–11. 10.3109/0284186X.2010.496793 20560860

